# The combined effects of microglia activation and brain glucose hypometabolism in early-onset Alzheimer’s disease

**DOI:** 10.1186/s13195-020-00619-0

**Published:** 2020-04-30

**Authors:** Giacomo Tondo, Leonardo Iaccarino, Silvia Paola Caminiti, Luca Presotto, Roberto Santangelo, Sandro Iannaccone, Giuseppe Magnani, Daniela Perani

**Affiliations:** 1 School of Psychology, Vita-Salute San Raffaele University, Milan, Italy; 2 In Vivo Human Molecular and Structural Neuroimaging Unit, Division of Neuroscience, IRCCS San Raffaele Scientific Institute, Milan, Italy; 3 Memory and Aging Center, Department of Neurology, Weill Institute for Neurosciences, University of California, San Francisco, CA USA; 4 Nuclear Medicine Unit, San Raffaele Hospital, Milan, Italy; 5 Department of Neurology and INSPE, San Raffaele Scientific Institute, Milan, Italy; 6 Clinical Neuroscience Department, San Raffaele Turro Hospital, Milan, Italy

**Keywords:** Positron emission tomography, Early-onset Alzheimer’s disease, [^18^F]-FDG PET, [^11^C]-(R)-PK11195 PET, Microglia activation

## Abstract

**Background:**

Early-onset Alzheimer’s disease (EOAD) is characterized by young age of onset (< 65 years), severe neurodegeneration, and rapid disease progression, thus differing significantly from typical late-onset Alzheimer’s disease. Growing evidence suggests a primary role of neuroinflammation in AD pathogenesis. However, the role of microglia activation in EOAD remains a poorly explored field. Investigating microglial activation and its influence on the development of synaptic dysfunction and neuronal loss in EOAD may contribute to the understanding of its pathophysiology and to subject selection in clinical trials. In our study, we aimed to assess the amount of neuroinflammation and neurodegeneration and their relationship in EOAD patients, through positron emission tomography (PET) measures of microglia activation and brain metabolic changes.

**Methods:**

We prospectively enrolled 12 EOAD patients, classified according to standard criteria, who underwent standard neurological and neuropsychological evaluation, CSF analysis, brain MRI, and both [^18^F]-FDG PET and [^11^C]-(R)-PK11195 PET. Healthy controls databases were used for statistical comparison. [^18^F]-FDG PET brain metabolism in single subjects and as a group was assessed by an optimized SPM voxel-wise single-subject method. [^11^C]-PK11195 PET binding potentials were obtained using reference regions selected with an optimized clustering procedure followed by a parametric analysis. We performed a topographic interaction analysis and correlation analysis in AD-signature metabolic dysfunctional regions and regions of microglia activation. A network connectivity analysis was performed using the interaction regions of hypometabolism and [^11^C]-PK11195 PET BP increases.

**Results:**

EOAD patients showed a significant and extended microglia activation, as [^11^C]-PK11195 PET binding potential increases, and hypometabolism in typical AD-signature brain regions, i.e., temporo-parietal cortex, with additional variable frontal and occipital hypometabolism in the EOAD variants. There was a spatial concordance in the interaction areas and significant correlations between the two biological changes. The network analysis showed a disruption of frontal connectivity induced by the metabolic/microglia effects.

**Conclusion:**

The severe microglia activation characterizing EOAD and contributing to neurodegeneration may be a marker of rapid disease progression. The coupling between brain glucose hypometabolism and local immune response in AD-signature regions supports their biological interaction.

## Introduction

The pathological hallmarks of Alzheimer’s disease (AD) include the extracellular beta-amyloid plaques and the intracellular neurofibrillary tangles, associated with neuronal and synaptic loss [1]. In the last decade, neuroinflammation mediated by microglia has been recognized to play a significant role in neurodegeneration and in AD pathogenesis [2]. However, whether neuroinflammation occurs as a causative or consequential factor in neurodegenerative processes is still an unsolved question [3].

Early-onset Alzheimer’s disease (EOAD) is an AD subtype characterized by onset of symptoms before the age of 65 years, which accounts for about 5% of AD cases [4]. The clinical and pathological features of EOAD significantly differ from those of late-onset AD (LOAD), thus suggesting a possible distinct pathophysiology [5–7]. EOAD patients have a greater clinical severity and a faster disease progression than LOAD [8–11]. EOAD clinical presentation is atypical in more than one third of cases, manifesting with impairment of executive functions, visuo-spatial abilities, or language, in addition to the characteristic episodic memory impairment [12]. Clinical symptoms reflect the presence of more extensive pathology, including a higher number of amyloid and tau deposits, greater synaptic loss, and brain atrophy [13–15].

Positron emission tomography (PET) imaging allows an in vivo evaluation of both neurodegeneration and neuroinflammation, thus providing evidence for pathophysiology in neurodegenerative diseases. [^18^F]-Fluorodeoxyglucose (FDG) PET is a topographical marker of brain glucose metabolism well representing the pattern of neurodegeneration in AD, in AD variants, and other neurodegenerative conditions [16–18], even in the prodromal phases [19–22]. The characteristic brain hypometabolism involving the associative temporo-parietal cortices, the precuneus and posterior cingulate cortex, is the distinctive dysfunctional pattern of AD since the early stage of the disease. In EOAD, [^18^F]-FDG PET imaging studies showed, at a comparable level of clinical severity, a greater brain hypometabolism than in LOAD [5, 23–25] and an extension of the hypometabolism in frontal and occipital regions in atypical presentations [26, 27]. More recently, [^18^F]-FDG PET measures have been used for the evaluation of brain metabolic connectivity [28]. [^18^F]-FDG PET brain glucose consumption reflects neuronal communication signaling, both locally and between distant brain regions, and is closely associated with functional connectivity [29]. Metabolic connectivity analysis relies on the assumption that regions whose metabolism is correlated are functionally interconnected [30]. The study of metabolic connectivity in AD has provided insights into the early changes in synaptic activity, by showing specific metabolic disconnections in AD dementia as well as in the prodromal phases [31, 32]. EOAD and LOAD showed distinct network features in comparison with healthy controls, with EOAD characterized by a more extensive and progressive alterations of connectivity [33].

Several tracers allow the in vivo study of neuroinflammation and, among these, the most used and validated is [^11^C]-(R)-PK11195 (1-(2-chlorophenyl)-N-methyl-N-(1-methylpropyl)-3-isoquinoline carboxamide). [^11^C]-(R)-PK11195 PET provides measures of microglial activation associated with mitochondrial overexpression of the 18 kDa translocator protein (TSPO), formerly known as peripheral benzodiazepine receptor [34]. Microglia are the resident immunocompetent cells of the central nervous system, activated in response to a variety of stimuli and in different pathological conditions, including neurodegeneration [35]. [^11^C]-(R)-PK11195 PET has been adopted in several studies involving AD cohorts, showing an increased tracer binding especially in the temporo-parietal, occipital, and cingulate cortex [36]. The relationship between microglia activity and glucose metabolism in AD was investigated combining [^11^C]-(R)-PK11195 and [^18^F]-FDG PET imaging, reporting a significant correlation between increased microglia activation and reduced glucose metabolism, both at baseline and over time. This finding suggests that neuroinflammation throughout the AD pathogenetic process is associated with synaptic dysfunction and glucose hypometabolism [37, 38]. Notably, studies employing a second generation TSPO tracer, [^11^C]-PBR28, in both EOAD and LOAD, reported a significantly higher tracer binding in frontal and parietal regions at baseline and over time, correlating with clinical severity [39], as well as a greater annual [^11^C]-PBR28 binding increase in temporo-parietal regions [40].

To date, the relationship between microglia activation and glucose hypometabolism in EOAD has not been investigated. Assessing the relationship between neuroinflammation and synaptic dysfunction in EOAD may contribute to the understanding of its pathophysiology and, possibly, to the selection of subjects for clinical trials. In our study, we hypothesized that microglia activation may play a significant role in EOAD neurodegeneration. Thus, we measured the correlation between microglia activation and glucose hypometabolism by using both [^11^C]-(R)-PK11195 and [^18^F]-FDG PET in single EOAD patients and comparing the results with a normal control database. Crucially, we also investigated the effect of metabolism-microglia spatial interaction in the modulation of network metabolic connectivity in the EOAD group.

## Methods

### Participants

Twelve patients with dementia, aged less than 65 years at disease onset, entered the study. They were admitted in the period from 2015 to 2017 to the Departments of Neurology and the Department of Rehabilitation and Functional Recovery, San Raffaele Hospital, Milan, Italy. All participants underwent a standard neurological evaluation by neurologists expert in evaluating dementia. Mini Mental State Evaluation (MMSE) and standard neuropsychological tests assessing memory, executive functions, language, and visuo-spatial abilities were obtained in all patients. Participants underwent blood analysis, lumbar puncture for cerebrospinal fluid analysis, brain MRI, and both [^18^F]-FDG PET and [^11^C]-(R)-PK11195 PET. The PET scans were performed at the Nuclear Medicine Unit, San Raffaele Hospital, Milan, Italy.

The study was approved by the San Raffaele Hospital Ethical Committee. We obtained informed written consent from patients and patient designated informants in accordance with the Declaration of Helsinki.

### PET imaging acquisition

The [^18^F]-FDG PET studies were acquired with a Discovery STE (GE Medical System, Milwaukee, WI) multi-ring PET tomography (PET-CT) system. Before radiopharmaceutical injection, patients were fasted for at least 6 h and their blood glucose level was < 120 mg/dl. Scans were acquired in resting state, in a dark set with patients lying with closed eyes, starting 45 min after injecting 185–250 MBq of [^18^F] FDG via a venous cannula. Image reconstruction was performed using an ordered subset expectation maximization (OSEM) algorithm. PET images were corrected for attenuation with a low-dose CT acquired contextually. Scatter correction was performed on all scans with the integrated software.

[^11^C]-(R)-PK11195 PET scans were performed on multi-ring PET homographs, either PET-CT Discovery LS or Discovery 690 general electric medical system (GEMS), injecting a dose of 380 ± 37 MBq of [^11^C]-(R)-PK11195. [^11^C]-(R)-PK11195 synthesis was performed in the Cyclotron Unit of the Nuclear Medicine of the San Raffaele Hospital as previously described [41], obtaining a radiochemical and chemical purity > 95%. Acquisition protocol included a dynamic PET scan of 15 frames lasting 58 min (6× 30 s/2× 1 min/1× 3 min/3× 5 min/2× 10 min/1× 15 min). PET data were corrected for attenuation artifacts, radioactive decay, and scatter. For each scan, movement correction was executed by realigning individual frames over time using the Statistical Parametric Mapping (SPM) 5 software (http://www.fil.ion.ucl.ac.uk/spm/software). [^11^C]-(R)-PK11195 PET scans of nine healthy controls (HC) (mean age 43.6 ± 11.2 years) were included for statistical comparison.

### Imaging processing and data analysis

#### [^18^F]-FDG-PET data processing

Data analysis was performed according to standardized and validated procedures [17, 42]. [^18^F]-FDG PET images were normalized at a single subject level using a dementia specific SPM FDG PET template [42], then smoothed with 8 mm full width at half maximum (FWHM) Gaussian kernel. Each patient scan was tested for relative hypometabolism by means of a two-sample *t* test implemented in SPM12, based on validated procedure in which the single image is compared on a voxel-by-voxel basis with a large normal database (*N* = 112) of [^18^F]-FDG PET images [17], including age as covariate. We operated proportional scaling to eliminate inter-subject global variation in PET intensities. In the resulting SPM t-maps, a threshold was set at *P* < 0.05 family wise error (FWE) corrected for multiple comparisons at the voxel level, considering significant clusters containing more than 100 voxels. We assessed EOAD hypometabolism pattern at the group level considering the same group of controls used for the single-subject analysis, performing a two-sample *t* test (EOAD group vs. HC group) covarying for age as a nuisance factor, setting significant threshold at 0.001 uncorrected, and considering significant clusters containing more than 100 voxels.

#### [^11^C]-(R)-PK11195-PET data processing

[^11^C]-(R)-PK11195 binding potentials (BP_s_) were estimated adopting a receptor parametric mapping (RPM) procedure, requiring a pre-set reference region [43]. Given the heterogeneous distribution of [^11^C]-(R)-PK11195 across the whole brain, hindering the delineation of a defined reference region, clustering methods have been proposed for PET studies of microglial activation. In our study, all the images were analyzed using the curve distance clustering algorithm (CDCA) [44], an adaptation of the validated SuperVised Clustering Algorithm [45]. The CDCA estimates the similarity of the time activity curve (TAC) of each voxel with four predefined TACs (tracer delivery in blood, white matter, grey matter with non-specific binding and high specific binding), allowing the selection of a cluster of voxels where the tracer kinetic is devoid of specific uptake. This pseudo-reference region can be used for the subsequent parametric analysis [44]. The clustering maps obtained with the CDCA method were warped to the standard Montreal Neurological Institute (MNI) space, then smoothed with 8 mm full width at half maximum (FWHM) Gaussian kernel. We explored voxel-wise statistical differences between the EOAD group and 9 healthy controls group at single-subject and group levels. Analyses were run with SPM12 covarying for age as a nuisance factor setting statistical threshold at *P* < 0.01 (uncorrected for multiple comparisons), considering significant those clusters containing more than 100 voxels.

#### Interaction analysis

To estimate the spatial interaction between hypometabolism and activated microglia, we considered binary masks of brain regional hypometabolism and microglial activation obtained from EOAD group vs. control groups in the voxel-wise comparisons. We obtained an interaction mask with IMcalc in SPM software applying the following formula:

Interaction mask = [^18^F]-FDG hypometabolism × [^11^C]-(R)-PK11195 microglial activation

We extracted cerebral glucose metabolic rate (rCMRG1c) uptake and [^11^C]-(R)-PK11195 BP from the interaction mask for each EOAD patient.

#### Correlation analysis

Correlation between [^18^F]-FDG hypometabolism and [^11^C]-(R)-PK11195 BP was investigated using Spearman’s rho correlation (*P* < 0.05). Statistical analysis was performed using IBM SPSS 22 (Statistical Package for Social Sciences software, IBM, Armonk, NY, USA).

#### Brain network analysis

A recent study investigating the association between [^11^C]-(R)-PK11195 binding increase and functional connectivity in AD patients showed a strong association between higher levels of neuroinflammation and abnormal connectivity, correlating with worse cognitive dysfunction [46]. To examine whether the spatial interaction between hypometabolism and microglia activation might affect large-scale metabolic connectivity in EOAD, we performed seed-based intercorrelation analysis, a validated method to explore metabolic connectivity using [^18^F]-FDG PET [47]. We used the interaction mask, previously obtained (see the “Interaction analysis” section*)*, as the seed volume of interest in order to perform the SPM voxel-wise interregional correlation analysis. Metabolic connectivity analysis was also performed in a group of 20 age- and sex-matched healthy controls (mean age 61.3 ± 3.5 years) for comparison.

## Results

Twelve patients met clinical diagnostic criteria for probable early-onset AD [48]. Considering clinical, neuropsychological, imaging, and CSF data, four patients fulfilled the criteria for typical AD (tAD), six patients fulfilled the criteria for frontal AD (fAD) variant, and two patients were diagnosed as posterior cortical atrophy (PCA) variant [49]. Table 1 shows patients demographic and neuropsychological data.

**Table 1:** Demographic and neuropsychological features in single EOAD patient

	EOAD1	EOAD2	EOAD3	EOAD4	EOAD5	EOAD6	EOAD7	EOAD8	EOAD9	EOAD10	EOAD11	EOAD12
Age, years	68	55	56	64	58	59	64	58	54	56	63	66
Education, years	5	13	13	8	13	8	8	8	8	13	8	5
Sex	F	M	M	F	F	M	F	F	M	F	F	M
Disease duration, years	5	1	1	3	2	1	1	5	1	2	3	2
Diagnosis	tAD	tAD	tAD	tAD	fAD	fAD	fAD	fAD	fAD	fAD	PCA	PCA
MMSE	**20**	**21**	**17**	**22**	**18**	**10**	**12**	**18**	**17**	**22**	**21**	**21**
Token test	31	31	**23.5**	29	**23**	**11.5**	**19.25**	**22.25**	33	31	27.5	30.5
PVF	27	**10**	**7**	29	**16**	**6**	**3**	**13**	22	31	56	44
SVF	30.1	**8**	**18.5**	42.8	**12**	**16**	**8**	**13**	45.5	44	29.2	59.2
Span forward	6.39	**4.84**	**2.83**	5.13	5.75	6.13	**2.92**	5.04	**2.92**	5.83	4.13	6.02
Span backward	4.53	**< 3**	**< 3**	3.19	**< 3**	**< 3**	**< 3**	3.1	**< 3**	3.79	4.19	**< 3**
RAVLT IR	**22.1**	**14.8**	**13.8**	**24.3**	**10**	**UT**	**UT**	**0**	36	41	**21.5**	32.3
RAVLT DR	**0**	**0**	**0**	**0**	**1.5**	**UT**	**UT**	**0**	**4.2**	**4**	**3**	**3.6**
ROF recall	**8**	**UT**	**0**	**8.5**	**5.25**	**UT**	**0**	**5**	**5.75**	11.75	**0**	**7.5**
ROF copy	**9**	**UT**	**0**	**23**	**13.25**	**3**	**7.25**	28.5	29.25	**14.75**	**0**	**24.25**
CDT	**1**	10	**2**	**5**	**5**	**0**	**0**	**0**	10	**7**	**0**	10
Attentive Matrix	36	**UT**	**12.5**	31.25	**23**	**UT**	**17.5**	**23**	**22**	49	34.25	**23**
Stroop time	**33.5**	28	**44.25**	**36.75**	29.25	**UT**	**UT**	**UT**	**46.8**	**46.17**	**44.25**	27
Stroop errors	**9.5**	3.5	**13**	**11.25**	**26.5**	**UT**	**UT**	**UT**	5	0	5.25	2.5
Raven PM	19.5	**UT**	**14**	19	**UT**	**UT**	**UT**	21.5	26.5	22	**12**	31.5

*EOAD* early-onset Alzheimer disease, *AD* Alzheimer disease, *tAD* typical AD, *fAD* frontal Alzheimer disease, *PCA* posterior cortical atrophy, *MMSE* mini mental state examination, *PVF* phonological verbal fluency, *SVP* semantic verbal fluency, *RAVLT* Rey auditory verbal learning task, *IR* immediate recall, *DR* delayed recall, *ROF* Rey-Osterrieth figure, *CDT* clock drawing test, *Raven PM* Raven Progressive Matrices, *UT* untestable

For neuropsychological assessment, pathological scores are reported in bold

### [^18^F]-FDG PET imaging findings

**Fig. 1:**
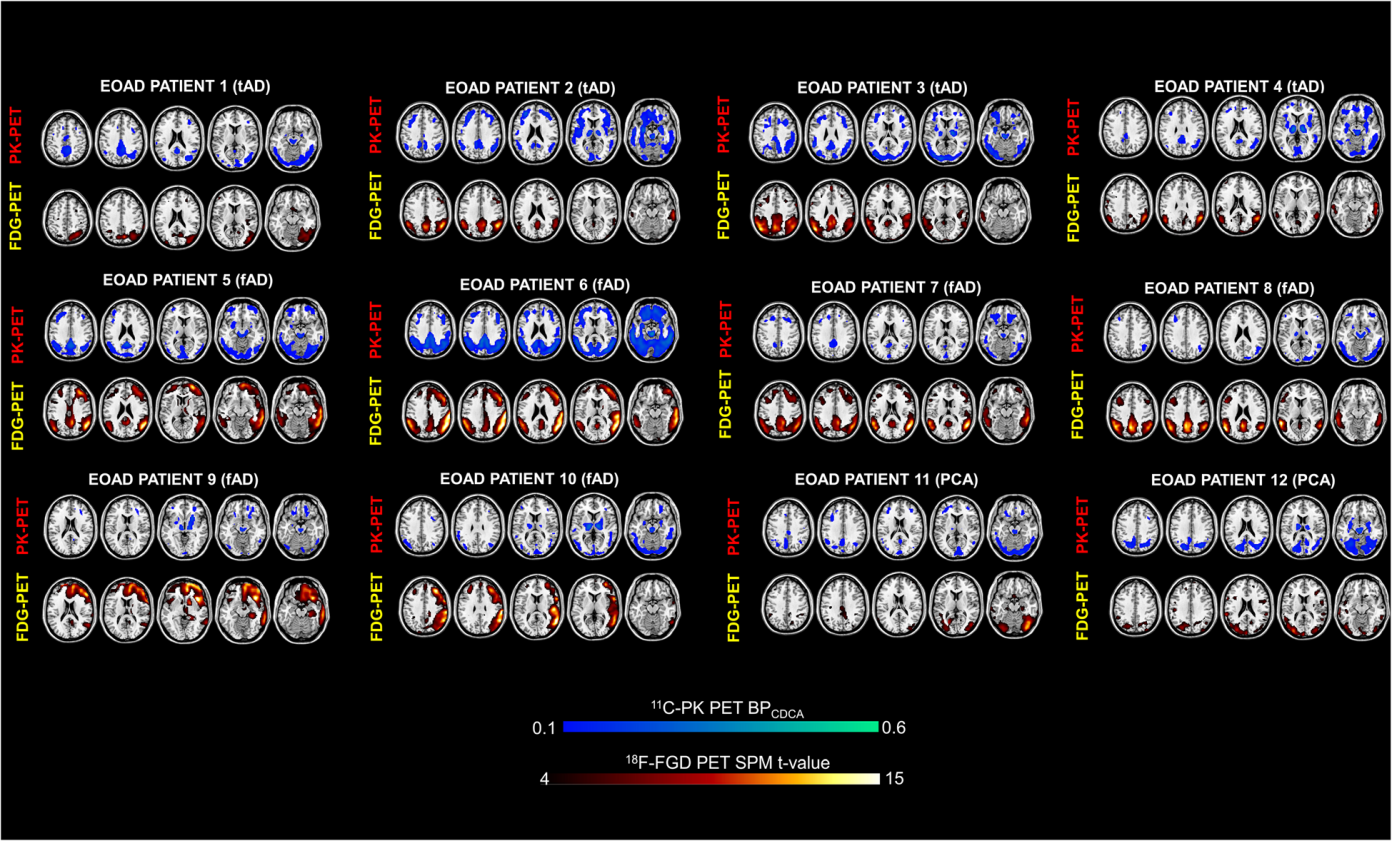
Patterns of [^18^F]-FDG PET brain hypometabolism and [^11^C]-PK 11195 PET binding potential in single individuals. tAD, typical Alzheimer disease; fAD, frontal AD variant; PCA, posterior cortical atrophy variant. Color scales for FDG and PK levels of significance

**Fig. 2:**
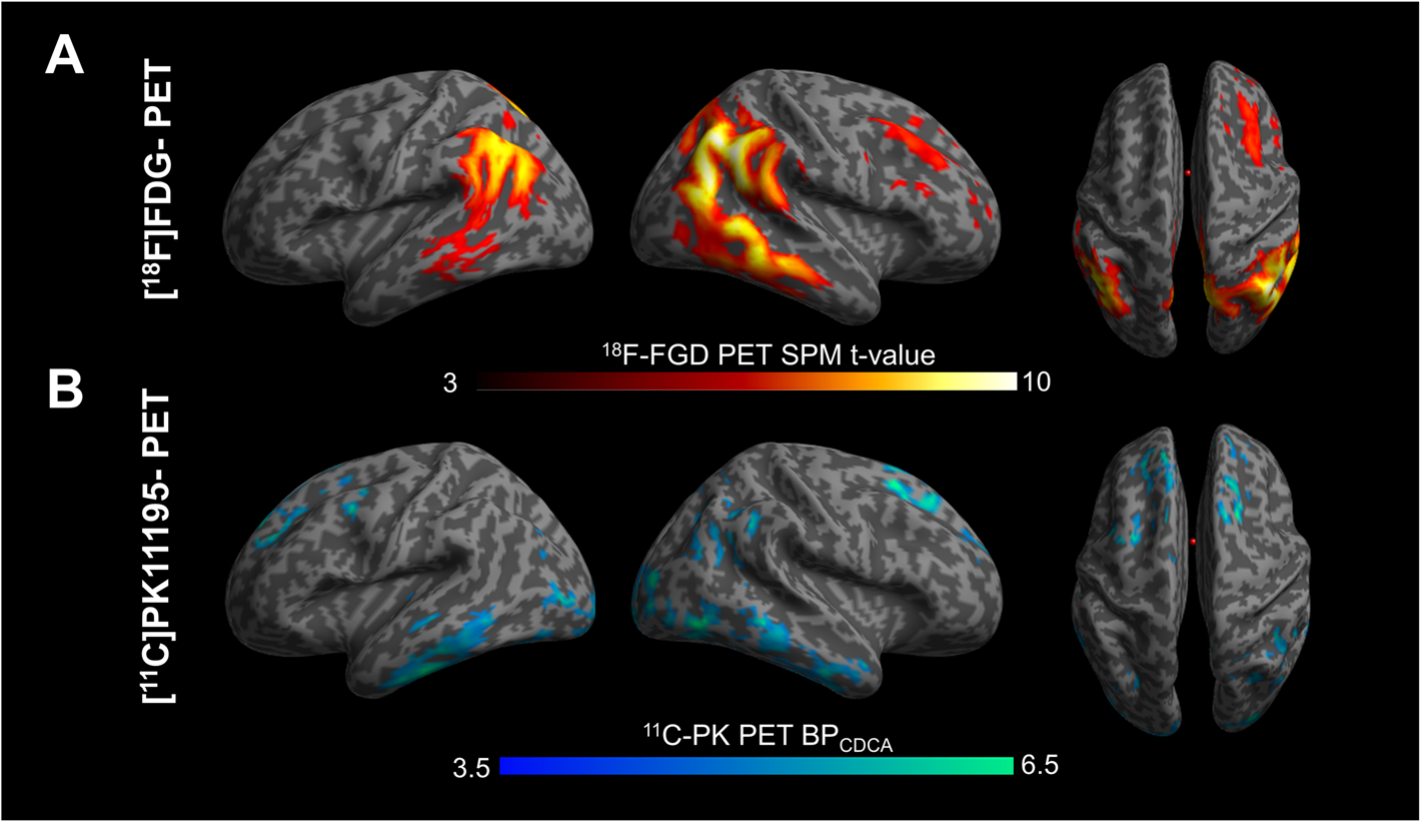
[^18^F]-FDG PET and [^11^C]-PK 11195 PET group analysis. The commonalities in the group comparison between early-onset Alzheimer’s disease (EOAD) patients and healthy controls. **a** The pattern of brain hypometabolism and **b** the increased [^11^C]-(R)-PK11195 binding potentials in the EOAD group. Results thresholded at *p* < 0.01 uncorrected*.* Color scales for [^18^F]-FDG and [^11^C]-PK 11195 levels of significance

The single subject procedure showed a consistent pattern of hypometabolism involving temporo-parietal regions, mainly in the right hemisphere, in all patients, with a variable involvement of occipital and frontal regions. A specific hypometabolic pattern of distribution was recognizable according to the clinical phenotype, namely frontal involvement in fAD patients and occipital hypometabolism in PCA patients (see Fig. 1). The single case presentation is included in the Additional file. The [^18^F]-FDG PET group analysis confirmed the presence of marked hypometabolism in temporo-parietal regions (see Fig. 2).

### [^11^C]-(R)-PK11195-PET imaging findings

The evaluation of the single subject [^11^C]-(R)-PK11195 BP maps showed a variable and significant BPs increases in all EOAD patients, with major clusters of activation in temporal, parietal, occipital, and frontal regions (see Fig. 1). At a group level, there was a significant difference between regional [^11^C]-(R)-PK11195 uptake in EOAD compared to HC bilaterally, in the middle inferior temporal gyrus, in the superior frontal gyrus, in the occipital gyrus, in the left precentral gyrus and left calcarine cortex, and in the right angular gyrus and right precuneus (see Fig. 2).

The single case presentation of clinical features, [^18^F]-FDG PET imaging findings, and increased [^11^C]-(R)-PK11195 BPs are detailed in the Additional file.

### Interaction analysis

The interaction analysis identified a spatial overlap in the right hemisphere temporo-parietal regions, namely in the inferior temporal gyrus, the precuneus, the angular gyrus, and the inferior parietal lobule.

### Correlation analysis

**Fig. 3:**
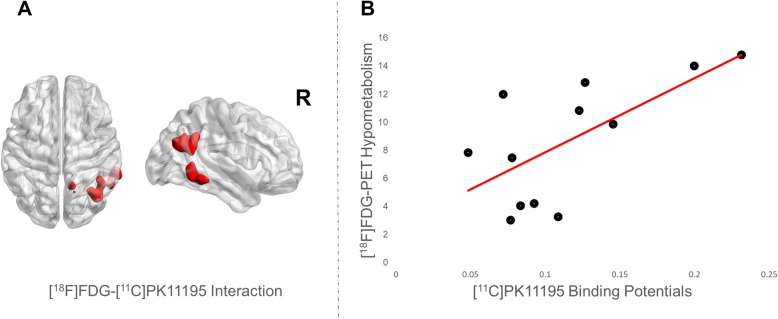
Regional interaction and correlation between hypometabolism and microglia activation. **a** Microglia activation and hypometabolism interaction (in red) overimposed on the surface-based map. **b** Correlation analysis obtained extracting cerebral glucose metabolism value and [^11^C]-PK 11195 binding potentials from the interaction regions (red line shows linear fitting). Correlation analysis indicates positive correlation between the two measures, meaning that higher microglia activation correlates with higher level of regional hypometabolism in the same regions

A higher microglial activation correlated with more severe hypometabolism in the same interaction regions (*r = 0.667, p 0.018*) (see Fig. 3).

### Network analysis

**Fig. 4:**
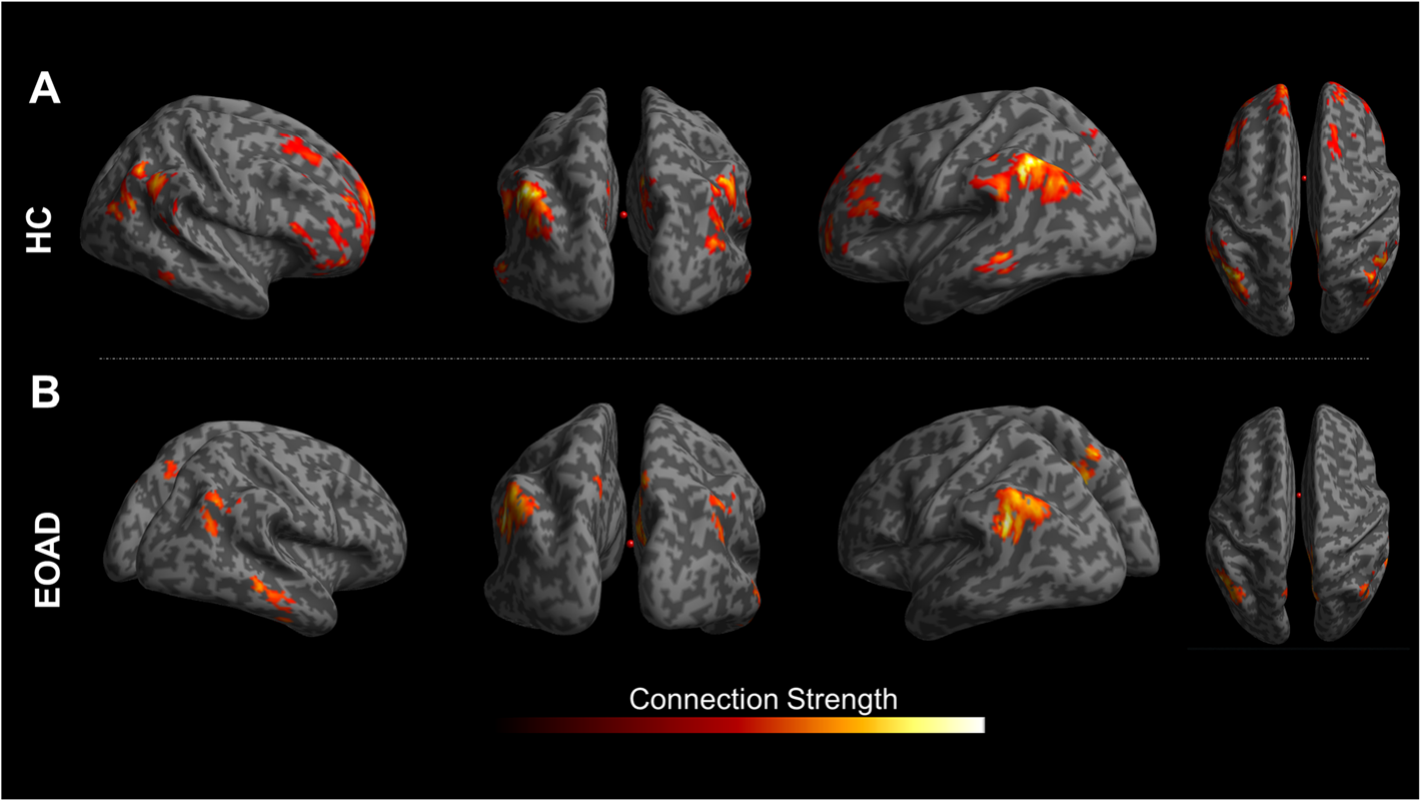
Network analysis. **a** The brain network connectivity within the temporo-parietal and frontal regions in healthy controls (HC) and **b** the brain network connectivity in EOAD patients showing the frontal disconnection. Connection strength bar represents the t-values obtained by means of statistical parametric mapping (SPM) interregional correlation analysis (see text for details). The network connectivity is overlaid on a surface-based map

Selecting as seed a region including the temporo-parietal interaction areas (see above), we identified an altered network in EOAD, mostly involving loss of connectivity between seed region and frontal cortex, in comparison with healthy subjects (see Fig. 4).

## Discussion

This is the first study evaluating the relationship of brain glucose hypometabolism with microglia activation in a group of EOAD patients, using both [^18^F]-FDG and [^11^C]-(R)-PK11195 PET. We showed a significant and widespread microglial activation in EOAD compared to controls, which had a spatial concordance with hypometabolism, mainly in AD-signature regions, i.e., the temporo-parietal cortices, but involving also frontal and occipital regions. The latter finding might be related to the presence of frontal and occipital variants in our EOAD series. Consistent with previous data in EOAD, [^18^F]-FDG PET imaging analysis showed a typical hypometabolism involving temporo-parietal region in all patients, with a variable involvement of frontal and occipital regions according to the clinical phenotype [24–26]. Coherently, in all fAD patients, there was also an involvement of frontal and prefrontal cortices, while PCA patients showed a hypometabolic pattern involving extensively the occipital regions.

Several PET studies using the radiotracer [11C]-(R)-PK11195 showed that microglia activation may be an early phenomenon in AD [38, 50–52] and that an increased uptake of [^11^C]-(R)-PK11195 is related to the distribution of AD hypometabolism, involving in particular temporo-parietal and posterior cingulate regions [50, 52]. Moreover, a recent in vivo study showed higher microglia activity in EOAD than in LOAD [40]. The role of microglial activation in EOAD, however, remains a scarcely explored field. In this study, we showed an increased [^11^C]-(R)-PK11195 uptake in temporo-parietal, occipital, and frontal regions, as the common inflammatory pattern within the group. The increased [^11^C]-(R)-PK11195 uptake showed an inter-subject variability in the single-subject analysis (Fig. 1). Notably, the frontal regional involvement in patients with fAD variant, and the posterior regions in the patients with PCA variant, suggest that a different pattern of neuroimmune activation may exist according to the clinical phenotype, as reported also in a previous work using [^11^C]-PBR28, a second generation TSPO tracer [53].

The precise role of microglia in AD pathogenesis is still debated. In normal function, microglia activity is crucial in protection against pathologic protein accumulation, e.g., favoring also amyloid clearance [54]. On the other hand, a chronic activation of microglia is likely to contribute to neurodegeneration [3]. Although the mechanisms inducing a detrimental microglial activation and subsequent neurodegeneration are not fully understood, synaptic dysfunction and neuronal loss may be due to different pathologic processes, such as the failure in clearance of amyloid plaques and cellular debris, the release of pro-inflammatory cytokines [55] or to a direct neuronal damage and phagocytosis, which has been reported in living neurons of a mice model of tauopathy [56].

In our study, all patients showed a spatial concordance/interaction between microglial activation and hypometabolism in AD-signature regions, peaking in the right temporo-parietal cortex. Previous studies in AD showed a correlation between microglial activation and markers of neurodegeneration, such as glucose brain hypometabolism and atrophy, or AD pathology markers, such as amyloid load [37, 38, 51]. The positive correlation between [^11^C]-(R)-PK11195 BPs and hypometabolism SPM maps and the spatial interaction between the two biological markers support the hypothesis of a relationship between microglial activation and neuronal synaptic dysfunction. Previous findings support the role of increased [^11^C]-(R)-PK11195 BPs in neuronal damage, given its correlation with global cognitive severity [51].

Considering as seed the regional interaction of microglial activation and hypometabolism, we found a long-distance loss of connectivity between temporo-parietal seed and frontal cortex, in comparison with the integrity of the same network relating parietal, occipital, and frontal regions in healthy controls. Regional microglial activation might negatively affect long-distance network organization, thus representing a relevant pathological event contributing to the neuronal damage and faster clinical progression typical of EOAD. A recent study investigating microglial activation and functional connectivity in AD suggested that neuroinflammation may be involved in pathophysiological changes in network function underlying cognitive deficits [46]. Our study confirms these only findings, suggesting that microglial activation may represent an early detrimental response in EOAD, underpinning an ongoing pathology in functionally connected regions in addition to the local damage, all driven by the neuroinflammation processes.

Our findings are limited by the small number of patients and by the inability of [^11^C]-(R)-PK11195 PET to distinguish among different microglia populations. Nevertheless, [^11^C]-(R)-PK11195 PET has been widely used in neurodegenerative conditions, showing consistent results [36].

## Conclusions

Previous studies showed that EOAD patients have more severe clinical manifestations than LOAD [9] and a greater hypometabolism and connectivity dysfunction [27]. Our study crucially adds that microglial activation may strongly contribute to neuronal damage in EOAD, providing new insights in the understanding of the underlying pathophysiology processes. In our sample, all EOAD patients show a coupled microglial activation and reduced glucose hypometabolism in key neurodegeneration regions for AD, with correlations between the two biological markers. Hypometabolism and neuroinflammation may thus act in a dynamic way, priming a vicious circle leading to faster clinical decline.

## Supplementary information

**Additional file 1.**

## Data Availability

The datasets used and/or analyzed during the current study are available from the corresponding author on reasonable request.

## Abbreviations

AD: Alzheimer’s disease
EOAD: Early-onset Alzheimer’s disease
LOAD: Late-onset Alzheimer’s disease
tAD: Typical Alzheimer’s disease
fAD: Frontal Alzheimer’s disease
PCA: Posterior cortical atrophy
HC: Healthy controls
PET: Positron emission tomography
FDG: Fluorodeoxyglucose
TSPO: 18 kDa translocator protein
SPM: Statistical Parametric Mapping
CDCA: Curve distance clustering algorithm
TAC: Time activity curve
BP: Binding potential
