# Supplementary material for: The combined effects of microglia activation and brain glucose hypometabolism in early-onset Alzheimer’s disease
Source: Alzheimers Res Ther. 2020 Apr 30;12:50. doi: 10.1186/s13195-020-00619-0 (PMC7193377; doi:10.1186/s13195-020-00619-0)
Supplement: Supplementary file 1 — Additional file 1. [file 13195_2020_619_MOESM1_ESM.docx]

**The combined effects of microglia activation and brain glucose hypometabolism in early-onset Alzheimer disease**

**Additional file**

Case 1. (68-year-old woman) Typical AD (tAD). Onset with progressively worsening memory deficits, associated with visuo-spatial abnormalities and mood disorders, anxiety and depression. (MMSE=20/30). Neuropsychological assessment revealed reduced performances in long-term memory, executive and visuo-spatial tests. CSF examination showed low Aβ42 and high p-tau levels. [^18^F]-FDG PET SPM map showed a marked hypometabolism involving the right temporo-parietal cortex, namely the middle temporal gyrus, the angular gyrus, the superior parietal gyrus, the fusiform gyrus and the precuneus. [^11^C]-(R)-PK11195 PET revealed microglia activation involving the temporal regions, mainly on the right, and the parietal and the occipital regions bilaterally. The microglia activation and brain glucose hypometabolism showed high spatial concordance in temporo-parietal regions.

Case 2. (55 years-old man). tAD. Onset characterised by memory deficits (MMSE=21/30). At the neuropsychological evaluation disturbances in long and short-term memory, language and executive functions were evident. [^18^F]-FDG PET SPM map revealed hypometabolism in the temporo-parietal cortex, namely in the middle temporal gyrus, the inferior parietal lobule, the precuneus and the posterior cingulate cortex bilaterally. [^11^C]-(R)-PK11195 Binding Potentials (BPs) were high in temporo-parietal regions, involving also, to a lesser degree, occipital and frontal regions.

Case 3. (56-year-old man) tAD. Long term memory deficits at presentation. (MMSE=17/30). The neuropsychological assessment revealed deficits in several explored domains and especially in executive and attentive functions, in language, in verbal and visuo-spatial memory and in visuo-spatial functions. CSF analysis showed low Aβ42 and high p-tau and t-tau levels. [^18^F]-FDG PET SPM map revealed hypometabolism in temporo-parietal regions, specifically in the inferior and middle temporal gyrus, the inferior parietal lobule, the precuneus and the posterior cingulate cortex bilaterally. [^11^C]-(R)-PK11195 PET showed increase of BPs in the temporo-parietal cortex, in the occipital and in the posterior cingulate cortex. The spatial concordance between microglia activation and brain glucose hypometabolism was high in temporo-parietal regions and in the posterior cingulate cortex.

Case 4. (64-year-old woman) tAD. Onset characterised by progressive worsening of memory. (MMSE=22/30). Neuropsychological evaluation revealed long term memory impairment, executive deficits and reduced performance in visuo-spatial functions. CSF analysis revealed low levels of Aβ42 and high levels of t-tau and p-tau. [^18^F]-FDG PET SPM map revealed a significant temporo-parietal hypometabolism, involving the middle temporal gyrus and the angular gyrus bilaterally. [^11^C]-(R)-PK11195 PET showed microglia activation in temporo-occipital cortex, partly involving also parietal and frontal regions and subcortical structures.

Case 5. (58-year-old woman) Frontal AD variant (fAD). Two years before diagnosis she showed marked behavioural alterations and apathy and progressive worsening of memory functions. (MMSE=18/30). Neuropsychological evaluation was characterised by deficits executive functions, visuo-spatial memory, visuo-spatial functions and also language and reasoning problems. CSF analysis revealed low levels of Aβ42 and high levels of t-tau and p-tau. [^18^F]-FDG PET SPM map analysis showed a marked temporo-parietal and frontal hypometabolism. [^11^C]-(R)-PK11195 PET analysis showed significant frontal, temporo-parietal and occipital microglia activation, right predominant, with additional subcortical structures involvement. The two biomarkers showed a highly concordant spatial distribution in temporo-parietal and frontal regions mainly in the right hemisphere.

Case 6. (59-year-old male) Frontal AD (fAD) variant. Clinical onset characterised by behavioural disturbances, impulsiveness and irritability. (MMSE=10/30). Neuropsychological assessment revealed deficits in executive, reasoning, language and visuo-spatial functions. CSF examination showed low Aβ42 and high p-tau and t-tau levels. [^18^F]-FDG PET SPM map showed an extensive cerebral hypometabolism involving temporo-parietal and frontal regions, with right side prevalence. [^11^C]-(R)-PK11195 PET revealed a widespread bilateral microglia activation in frontal, temporal, parietal, and occipital regions. Microglia activation showed diffused spatial concordance with brain glucose hypometabolism, particularly in temporal, parietal and frontal cortex.

Case 7. (64-year-old woman) fAD variant. Onset one year before with language and memory disturbances, with hallucination and behavioural abnormalities such as violent and auto aggressive behaviour. (MMSE=12/30). Neuropsychological assessment revealed short and long-term memory, executive, language and visuo-spatial deficits. [^18^F]-FDG PET SPM map showed a severe hypometabolism in temporo-parietal regions (inferior and middle temporal gyrus, angular gyrus, precuneus, inferior parietal lobule) bilaterally, and in the prefrontal cortex. [^11^C]-(R)-PK11195 showed bilateral microglia activation in temporo-occipital and frontal regions and, in the precuneus bilaterally.

Case 8. (58-year-old woman) fAD variant. The disease presentation with depression, anxiety and language difficulties. (MMSE=18/30). The neuropsychological assessment showed abnormalities in executive, language, visuo-spatial, short term and long-term memory functions. [^18^F]-FDG-PET SPM map revealed brain hypometabolism in the middle temporal gyrus, inferior parietal lobule, precuneus, and posterior cingulate cortex, and in the inferior and middle frontal gyrus on the left. [^11^C]-(R)-PK11195 BPs were high in temporal, occipital regions and also, to a lesser degree, in frontal regions.

Case 9. (54-year-old man) fAD variant. Apathy and planning difficulties at the disease onset, with associated sweet craving, irritability and sexual disinhibition. (MMSE=17/30). The neuropsychological profile was characterised by attentional, executive and working memory deficits. CSF examination was abnormal for low Aβ42 levels and tau normal values. [^18^F]-FDG PET SPM map revealed diffuse hypometabolism involving the right hemisphere in the orbitofrontal, the dorso-lateral cortex, temporal cortex, angular gyrus and the precuneus, and the posterior cingulate cortex. [^11^C]-(R)-PK11195 BPs showed microglia activation in orbito-frontal cortex and subcortical structures. The topographical distribution of the two biomarkers was concordant mainly in the right frontal cortex and temporo-parietal cortex.

Case 10. (56-year-old woman) fAD variant. At the disease presentation she presented difficulties in planning and in daily activities, anxiety and irritability. (MMSE 22/30). Executive and visuo-spatial impairment were present at the neuropsychological evaluation. CSF analysis revealed low Aβ42 and high p-tau and t-tau levels. [^18^F]-FDG PET SPM map showed hypometabolism in temporo-parietal and frontal cortices, in the right hemisphere, involving the superior temporal gyrus, the angular and supramarginal gyrus, the superior frontal gyrus. [^11^C]-(R)-PK11195 PET analysis showed microglia activation in temporal and occipital regions.

Case 11. (63-year-old woman) Posterior Cortical Atrophy (PCA). Disease presentation characterised by memory and visual disturbances, anxiety and depression. (MMSE=21/30). Neuropsychological evaluation revealed deficits in long-term memory, visuo-spatial and visuo-perceptive functions (objects recognition deficit), plus alexia and executive functions deficit. CSF analysis revealed low levels of Aβ42 and high levels of p-tau. [^18^F]-FDG PET SPM map highlighted a marked hypometabolism involving occipital and temporal cortex bilaterally. [^11^C]-(R)-PK11195 PET analysis showed an increased BPs in occipital and temporal cortex. The latter findings are consistent with a shared topography of the two biomarkers.

Case 12. (66-year-old man) PCA. He had progressively worsening of memory disturbance. (MMSE=21/30). The neuropsychological assessment revealed severe deficit in verbal and visuo-spatial memory, in the visuo-spatial and in the visuo-perceptive abilities (complex figure copy and space perception deficit), and constructional dyspraxia. CSF analysis showed low Aβ42 and high p-tau levels. [^18^F]-FDG PET SPM map showed hypometabolism involving temporoparietal and occipital regions. [^11^C]-(R)-PK11195 BPs were increased showing a diffuse microglia activation in temporal, occipital and parietal regions bilaterally. The latter findings support a comparable topography of hypometabolism and microglia activation.
